# The serum activity of thioredoxin reductases 1 (TrxR1) is correlated with the poor prognosis in EGFR wild-type and ALK negative non-small cell lung cancer

**DOI:** 10.18632/oncotarget.23252

**Published:** 2017-12-14

**Authors:** Gong Chen, Qiong Chen, Fanxu Zeng, Liang Zeng, Haiyan Yang, Yi Xiong, Chunhua Zhou, Li Liu, Wenjuan Jiang, Nong Yang, Yongchang Zhang

**Affiliations:** ^1^ Departement of Medical Oncology, Lung Cancer and Gastrointestinal Unit, Hunan Cancer Hospital/Affiliated Cancer Hospital of Xiangya School of Medicine, Changsha 410013, China

**Keywords:** thioredoxin reductases 1, bio-marker, EGFR wild type and ALK negative non-small-cell lung cancer, CEA, prognosis

## Abstract

**Background:**

The thioredxin reductases 1 (TrxR1) is one of the major antioxidant and redox regulators in mammalian cells. Studies have shown that TrxR1 is over expressed in many malignancy diseases. However, few studies have evaluated the role of TrxR1 in non-small cell lung cancer (NSCLC).

**Methods:**

Serum levels of TrxR1 and CEA in 142 patients with EGFR wild type and ALK negative advanced NSCLC was measured by ELISA assay before first line standard doublet chemotherapy from June 2013 to February 2016 in Hunan Cancer Hospital. Clinical characteristics and Survival data were collected and analyzed according to serum TrxR1 levels.

**Results:**

No significant differences were founded from clinic pathological variables. With the cut-off value of 12U/mL, the lower serum TrxR1 activity patients had long progression-free survival (PFS) and overall survival (OS) compared with higher patients (PFS: 5.3m vs. 3.6m p=0.044, OS: 14.5m vs. 11m *p*<0.001). In subgroup, lower serum TrxR1 activity patients had long OS both in adenocarcinoma (ADC) (17m vs. 8m, *p*=0.003) and squamous cell carcinoma (SCC) (13m vs. 11m, *p*=0.035). While combining with TrxR1 activity and serum CEA concentrations, we founded that patients with lower serum TrxR1 activity and serum CEA concentrations had long OS compared with higher group patients (20m vs. 7m, *p*<0.001).

**Conclusions:**

Serum TrxR1 activity was not affected by clinic pathological variables. Measurement of serum TrxR1 activity might be an independent prognostic factor for EGFR wild type and ALK negative advanced NSCLC patients. Combination of serum TrxR1 activity and serum CEA concentrations need to be further profiled from bench to beside.

## INTRODUCTION

Lung cancer was the most frequently diagnosed cancer and the leading cause of cancer death both in our country and worldwide [1]. Non-small-cell lung cancer (NSCLC) accounts for at least 80% of all lung cancer cases, presenting as locally advanced disease in approximately 25-30% of cases and as metastatic disease in approximately 40-50% of cases [2]. The median overall survival (OS) is 4-6months platinum-based combination chemotherapy improves the quality of life as well as prolonging the survival of patients with advanced NSCLC [3]. During recent decades, with the development of chemotherapy, treatment of NSCLC with chemotherapy could prolong survival time of the patients and cost less than the supportive care treatment alone. Especially, in recent years, many molecular targeted drugs, such as gefitinib, erlotinib and crizotinib, have been successively applied in clinical use, and they bring about a substantial prolongation of survival life and improvement in life quality of those patients with advanced lung cancer [4, 5]. So far, only a few of factors are well recognized as prognostic indicators for lung cancer, including tumor size, tumor depth, lymph node metastasis (LNM), TNM stage and so on. Although there is some exploration about the biomarkers, such as CEA, we still have no powerful evidence to prove a reliable sensitivity and specificity factors to predict the prognosis of NSCLC patients.

The thioredoxin reductase (TrxR) is a selenium-containing enzyme part of the thioredoxin system responsible for regulating oxidative stress and redox signaling via reduction of disulfide bonds. The dimer, Thioredoxin Reductase 1(TrxR1), reduces thioredoxin and other substrates using NADPH and an FAD cofactor [6, 7]. TrxR1 involvement in protecting against oxidative stress and injury, regulation of cellular development and growth, and various other cellular processes make it an interesting target for studies of various cancers, AIDS, and other diseases. Humans express three different ozymes of thioredoxin reductase, isoform 1 is the cytosolic form, isoform 2 is the mitochondrial form, and isoform 3 is a testes specific form [8, 9]. The thioredoxin system is mainly composed of TrxR, Trx and NADPH; sustain several important thioredoxin-dependent pathways. The system involved in many central intracellular and extracellular processes including cell proliferation, the redox regulation of gene expression and signal transduction, protection against oxidative stress, anti-apoptotic functions, growth factor and co-cytokine effects, and regulation of the redox state of the extracellular environment [10–15]. TrxR1 plays an important role in regulating cancer cell growth, for example, by modulating the DNA binding activity of transcription factors, including nuclear factor p53, and glucocorticoid and estrogen receptors [16, 17]. TrxR1 is potentially important in conjunction with the onset of many diseases including inflammatory diseases, heart failure, cancer [18–21]. Many studies have shown that TrxR1 was overexpressed in a variety of primary tumors and its activity was highly relevant [22, 23].

In our previous study, we found that the expression of TrxR1 in NSCLC tissues was evidently stronger than that in their corresponding adjacent normal lung tissues and the difference was significant. Meanwhile we found that the expression of TrxR1 in adenocarcinoma and squamous cell carcinoma was 47% and 50% respectively (data no shown). Then we conducted a prospective study and investigated that the activity of TrxR1 in serum is significantly higher in 1122 cancer patients than 84 volunteers. In addition, the specificity of area under receiver operating characteristic curve (ROC) for serum TrxR1 was 95% when the cut-off value of TrxR1 was 12U/ml for discriminating NSCLC from healthy controls [24].

So, we conducted a retrospective study aims to prove that TrxR1 is a novel biomarker for prognosis, and we also want to evaluate the prognostic role when we combine TrxR1 with CEA. Our results showed that the evaluation of serum TrxR1 could be a valuable prognostic biomarker for NSCLC.

## RESULTS

### Clinical characteristics of patients

In this study, we recruited a total of 142 EGFR wild type and ALK negative NSCLC patients. The demographic, pathologic, and clinical information of the study subjects with gender, age, histology, TNM stage, smoking index, CEA levels included were displayed in Table 1. The patients under the age of 60 years accounted for 59.9% of all patients. The proportion of male gender accounted for 88.0% while the female was only 12%. At the time of diagnosis, 99 patients were at stage IV with distant metastases, 43 patients were at stage IIIB+IIIC. The histology of all recruited patients was adenocarcinoma or squamous cell carcinoma, accounted for 44.4% and 55.6% respectively. More than half of the patients had a long history of smoking with smoking index exceeded 400. Patient’s data were analyzed after a 3-year follow-up.

**Table 1:** Clinic pathological variables of NSCLC patients

Variables	NSCLC patients (n = 142) (%)
**Age, years**	
<60	85(59.9)
≥60	57(40.1)
**Gender**	
male	125(88.0)
female	17(12.0)
**Histology**	
ADC	63(44.4)
SCC	79(55.6)
**TNM stage**	
IIIB-IIIC	43(30.3)
IV	99(69.7)
**Smoking index**	
<400	38(26.8)
≥400	104(73.2)
**Serum CEA(ng/mL)**	
<3.4	73(51.4)
≥3.4	69(48.6)
**Chemotherapy regimen**	
PP	40(28.2)
TP	39(27.5)
GP	63(44.3)

### There was no association between serum TrxR1 activity and clinic pathological variables

We further evaluated the clinic pathological significance of the serum TrxR1 activity in NSCLC patients. The association between the serum TrxR1 activity and clinic pathological variables in NSCLC patients were summarized in Table 2. As displayed, the factors we analyzed included age, gender, histology, TNM stage, smoking index and serum CEA, no significant differences were founded from these clinic pathological variables to serum TrxR1 activity. These results indicated that serum TrxR1 activity may be not affected by usual clinic pathological variables.

**Table 2:** TrxR1 levels and characteristical variables in EGFR wild type and ALK negative NSCLC patients

Factors	Number	TrxR1 (U/ml) Median (95%CI)	*p*- value
**Age (years)**			0.344
<60	85	11.90(10.12~13.68)	
≥60	57	14.19(8.93~19.45)	
**Gender**			0.552
male	125	13.8(10.46~15.69)	
female	17	10.91(7.30~14.51)	
**Histology**			0.610
ADC	63	13.49(8.67~18.32)	
SCC	79	12.28(10.46~14.10)	
**TNM stage**			0.786
IIIB-IIIc	43	12.33(9.97~14.69)	
IV	99	13.03(9.82~16.24)	
**Smoking index**			0.358
<400	38	11.02(8.97~13.07)	
≥400	104	13.48(10.37~16.58)	
**Serum CEA(ng/Ml)**			0.816
<3.4	73	12.55(10.97~14.13)	
≥3.4	69	13.10(8.53~17.67)	

*p*<0.05 was considered statistically significant.

### Serum TrxR1 activity were an independent poor prognostic indicator for EGFR wild type and ALK negative NSCLC patients

**Figure 1:**
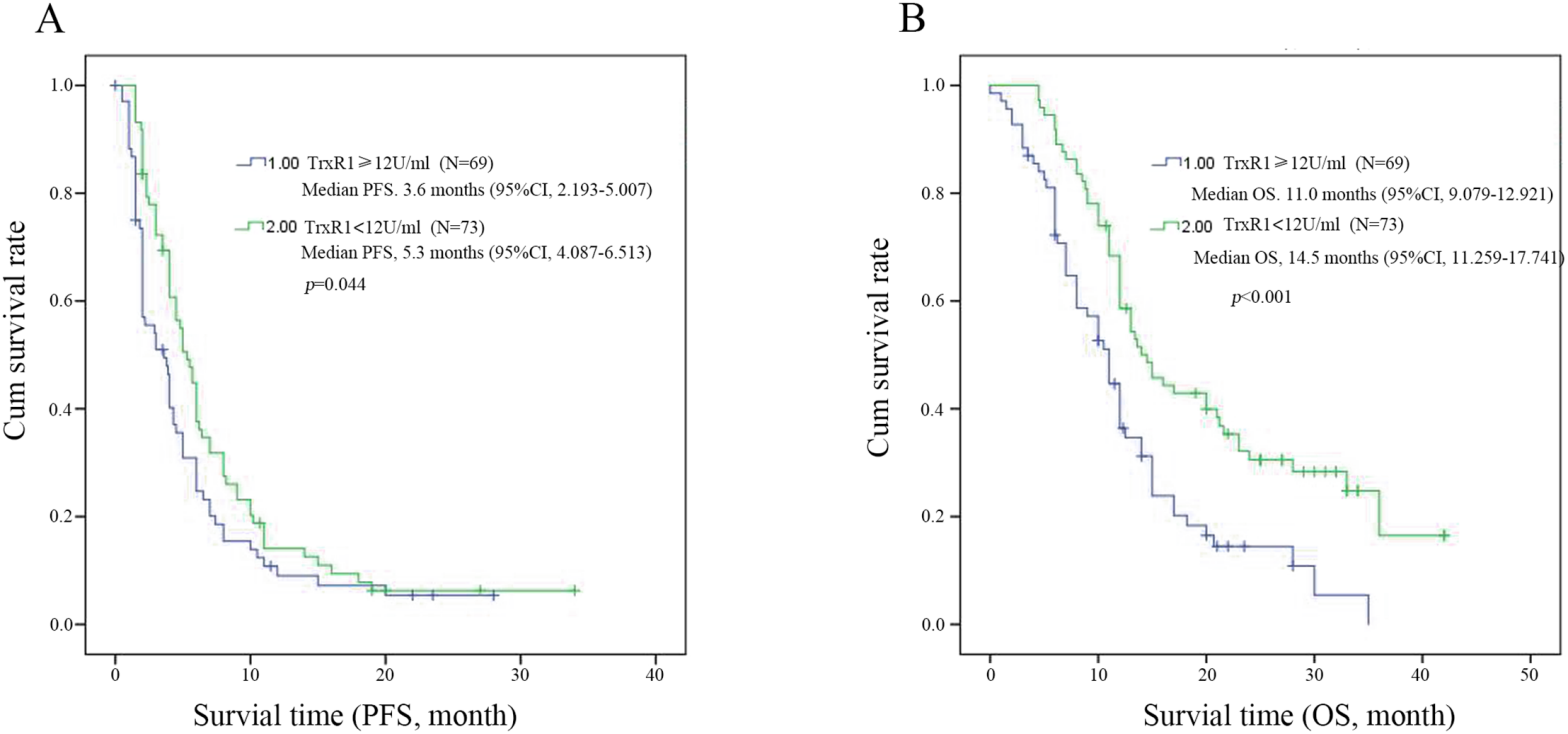
Kaplan-Meier survival curves for PFS **(A)** and OS **(B)** in all patients with high and low level activity TrxR1 NSCLC. Log-rank test determined that the PFS and OS in high TrxR1 group were shown in the low and high TrxR1 group, p<0.05 was considered statistically significant.

**Figure 2:**
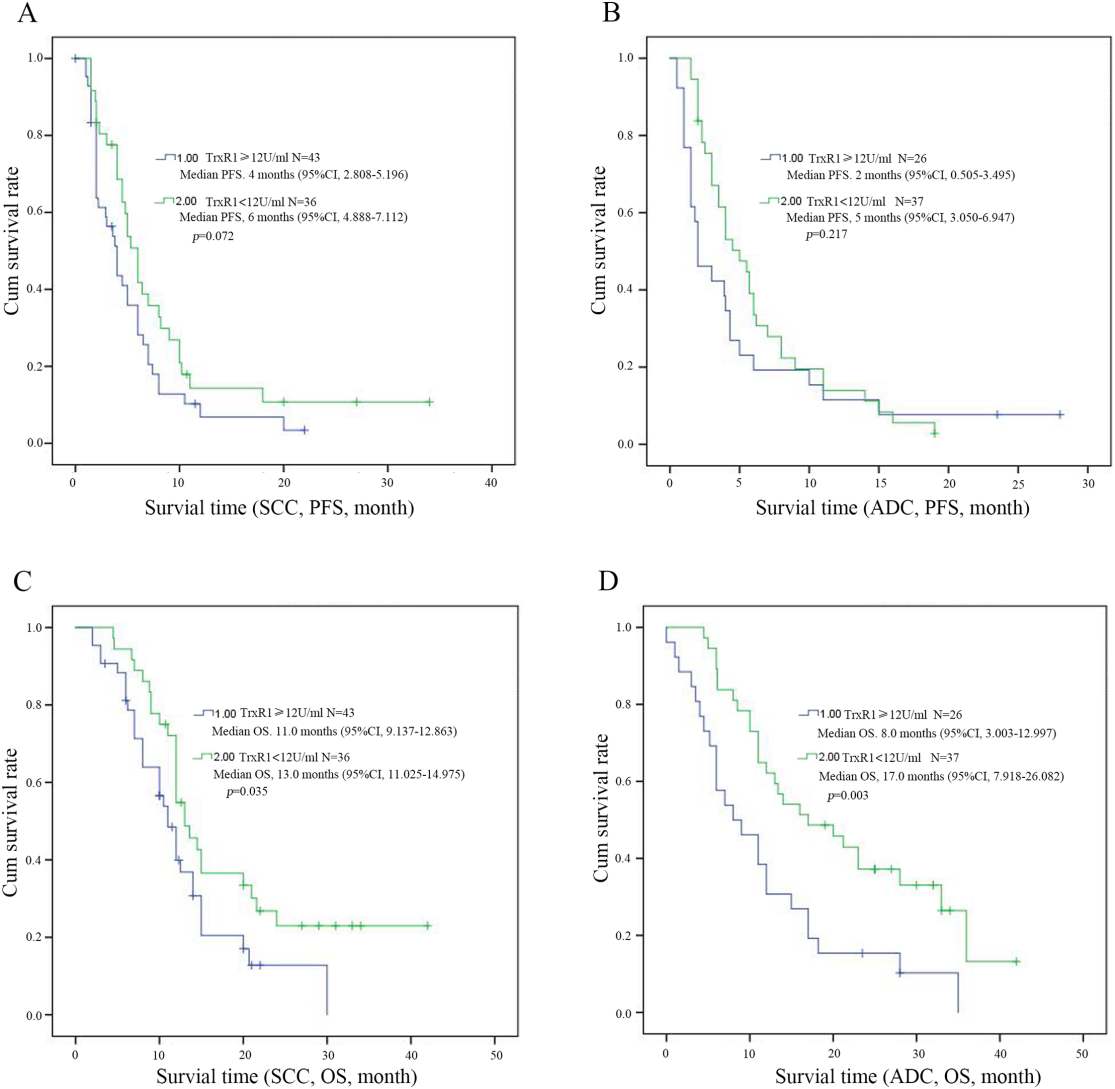
Kaplan-Meier survival curves for PFS and OS in patients with high and low level TrxR1 activity of NSCLC (**A**, **C** for SCC, **B** and **D** for ADC). The survival time of patients with different histology were significantly shown in low and high TrxR1 activity subgroup, p<0.05 was considered statistically significant.

**Figure 3:**
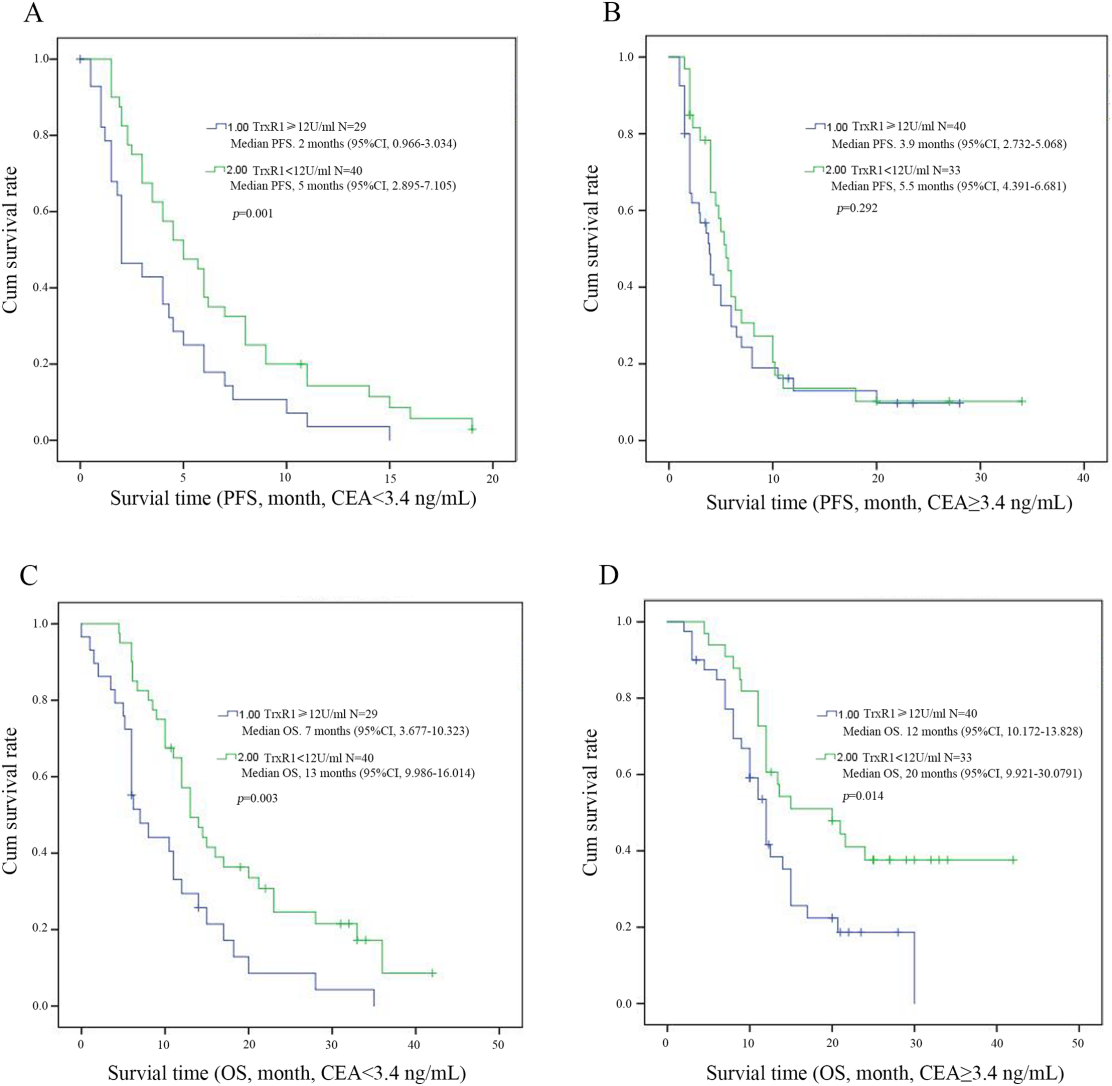
Kaplan-Meier survival curves for PFS and OS in patients with different levels of serum TrxR1 activity through CEA concentration **(A** and **C)** show the low level CEA group (CEA<3.4 ng/mL); **(B** and **D)** show the high level CEA group (CEA{greater than or equal to}3.4 ng/mL). The survival time of PFS (A) and OS (B) were shown in patients of low TrxR1 activity subgroup regardless of the low level of serum CEA concentration. The survival time of OS (D), and the PFS (B) were shown in patients of low TrxR1 activity subgroup regardless of the high level of serum CEA concentration, p<0.05 was considered statistically significant.

To evaluate the prognostic significance of the serum TrxR1 activity, we used serum TrxR1 cut-off value 12 U/ml, which was calculated from previous study, as a threshold to partitioned 142 NSCLC patients into two groups, high serum TrxR1 activity group (TrxR1 ≥12 U/ml, n = 69) and low serum TrxR1 activity group (TrxR1 < 12 U/ml, n=73). Univariate analysis showed that serum TrxR1 activity were significantly correlated OS, however not PFS. The other clinic pathological variables analysis showed low smoking index and early stage have significant correlation with longer PFS (Table 3). In multivariate analysis, high serum TrxR1 activity was found to be significantly associated with a shorter OS and PFS. Early stage and low serum CEA had significant correlation with longer PFS and OS respectively (Table 4). Kaplan-Meier survival curves further demonstrate that NSCLC patients with low serum TrxR1 activity have longer PFS (5.3m vs. 3.6m, *p*=0.044, Figure 1A) and OS (14.5m vs. 11.0m, *p*<0.001, Figure 1B), compared to those with high serum TrxR1 activity. In subgroup analysis, PFS has an extended trend in patients with low serum TrxR1 activity, both in SCC(6m vs. 4m, *p*=0.072, Figure 2A) and ADC(5m vs. 2m, *p*=0.217, Figure 2B). Moreover, the OS of two different histology were significantly prolonged in low TrxR1 activity subgroup, 13m vs. 11m in SCC(*p*=0.035, Figure 2C) and 17m vs. 8m in ADC(*p*=0.003, Figure 2D). Besides, in subgroup of high serum CEA, low TrxR1 activity was correlated with longer PFS (5m vs. 2m, *p*=0.01, Figure 3A) and OS (13m vs. 7m, *p*=0.003, Figure 3C). While in subgroup of low serum CEA, only OS was prolonged (20m vs. 12m, *p*=0.014, Figure 3D). No significant difference was found in PFS (5.5m vs. 3.9m, *p*=0.292, Figure 3B).

**Table 3:** Univariate of variables considered for PFS and OS of NSCLC patients

Variables	PFS			OS		
	HR	95%CI	*p*-value	HR	95%CI	*p*-value
**Age**(< 60 vs. ≥60)	1.121	0.787-1.595	0.528	1.316	0.898-1.927	0.159
**Gender**(male vs. famale)	0.722	0.420-1.239	0.237	0.580	0.321-1.050	0.072
**Histology**(ADC vs. SCC)	1.147	0.808-1.627	0.443	0.896	0.612-1.313	0.573
**TNM stage**(IIIB+IIIC vs. IV)	2.016	1.400-2.903	<0.001	1.163	0.795-1.701	0.436
**Smoking index**(< 400 vs. ≥400)	0.647	0.443-0.945	0.024	0.658	0.429-1.010	0.055
**Serum CEA (ng/mL)**(<3.4 vs. ≥3.4)	0.780	0.549-1.108	0.165	0.726	0.497-1.060	0.097
**TrxR1**(< 12 vs. ≥12)	0.709	0.500-1.005	0.054	0.506	0.345-0.744	<0.001
**Chemotherapy regimen**(PP vs. TP vs. GP)	0.878	0.709-1.086	0.230	0.954	0.756-1.203	0.688

*p*<0.05 was considered statistically significant.

**Table 4:** Multivariate Cox analysis of variables considered for PFS and OS of NSCLC patients

Variables	PFS			OS		
	HR	95%CI	*p*-value	HR	95%CI	*p*-value
**Age**(< 60 vs. ≥60)	1.059	0.739-1.519	0.753	1.357	0.911-2.021	0.133
**Gender**(male vs. female)	0.855	0.427-1.712	0.659	0.630	0.293-1.351	0.235
**Histology**(ADC vs. SCC)	1.282	0.821-2.001	0.275	0.871	0.558-1.360	0.545
**TNM stage**(IIIB+IIIC vs. IV)	1.876	1.229-2.862	0.004	1.263	0.834-1.911	0.270
**Smoking index**(< 400 vs. ≥400)	0.646	0.376-1.109	0.113	1.001	0.552-1.816	0.998
**Serum CEA (ng/mL)**(<3.4 vs. ≥3.4)	0.882	0.585-1.331	0.551	0.619	0.406-0.943	0.026
**TrxR1**(< 12 vs. ≥12)	0.643	0.448-0.923	0.017	0.468	0.314-0.698	0.000
**Chemotherapy regimen**(PP vs. TP vs. GP)	0.935	0.744-1.174	0.562	0.999	0.791-1.260	0.990

*p*<0.05 was considered statistically significant.

### Combined prognostic value of the TrxR1 and CEA means better prognostic value in NSCLC

**Figure 4:**
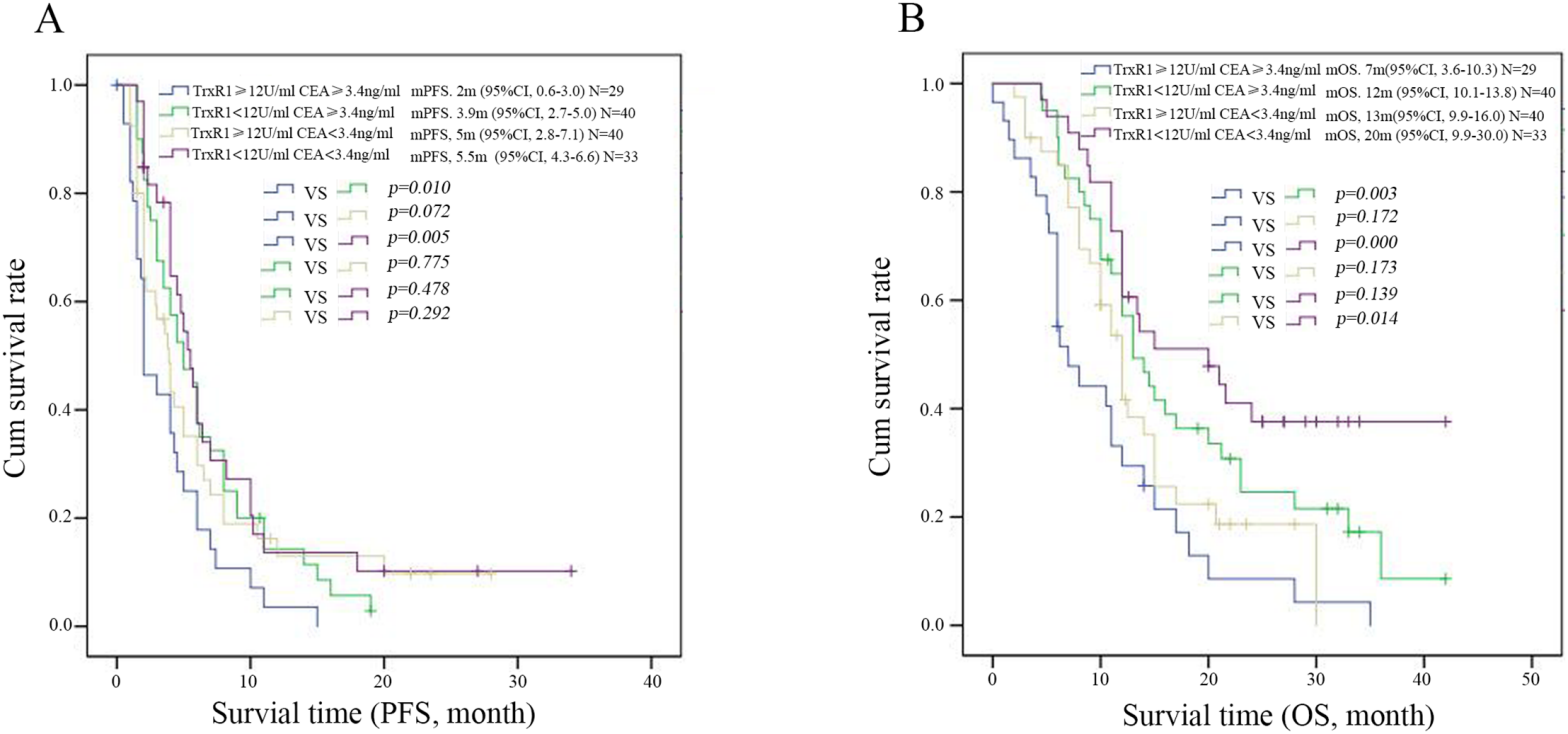
Kaplan-Meier survival curves for PFS **(A)** and OS **(B)** in patients with different levels of serum TrxR1 activity and CEA concentration. The PFS and OS were shown in patients of low TrxR1 activity and low CEA concentration subgroup than those in the high TrxR1 and CEA group, p<0.05 was considered statistically significant.

CEA level was one of the markers associated with lung cancer diagnosis and commonly used in clinic. We further analyzed the value of combined detection of serum CEA concentration and TrxR1 activity in the prognosis of patients. Serum CEA concentration and TrxR1 activity levels are shown in Table 5. By survival analysis, we founded that patients with low serum TrxR1 activity and CEA concentration have longer PFS (5.5m vs. 2m, *p*=0.005, Figure 4A) and OS (20m vs. 7m, *p*<0.001, Figure 4B), compared to those with high serum TrxR1 activity and CEA concentration.

**Table 5:** The distribution of serum TrxR1 activity and CEA concentration in NSCLC patients

		Serum TrxR1 activity (U/mL)	
		high (≥12)	low (<12)
**Serum CEA concentration(ng/mL)**	**high (≥3.4)**	**29**	**40**
	**low (<3.4)**	**40**	**33**

## DISCUSSION

TrxR1, as a crucial member of thioredoxin system, is mainly distributed in the cytoplasm and responsible for maintaining the pool of reduced and active Trx [19]. In our study, the prognosis value of serum TrxR1 activity in EGFR wild type, ALK negative NSCLC patients were evaluated.

We had analyzed the relationship between serum TrxR1 activity and seven unfavorable clinic pathological variables which we thought may have the potential influence on serum TrxR1 activity, however, no significant differences was founded. These results suggested that serum TrxR1 activity may be not affected by clinic pathological variables.

So far, only a few prognostic factors have been identified, such as, performance status, weight loss, disease stage, gender [25]. However, we cannot accurately predict the prognosis if only consider clinical parameters. Therefore, the discovery of reliable molecular prognostic factors of NSCLC patients is valuable. Previous studies had shown thioredoxin expression is associated with lymph node status and prognosis in early operable non-small cell lung cancer [26]; however the prognostic significance of TrxR1 in NSCLC is still unclear. In our study, survival analysis revealed the patients with low serum TrxR1 activity had a significant longer PFS and OS compared to those with high serum TrxR1 activity. In further subgroup analysis, whether in different histology or CEA concentration, low TrxR1 activity also associated with better prognosis. Moreover our results showed that the combined detection of TrxR1 and CEA had a better prognostic value than the use of a single index. Univariate analyses showed that increased TrxR1 activity in serum of NSCLC is significantly associated with the overall survival rate. Moreover, multivariate analysis demonstrated that TrxR1 activity is an independent risk factor in the prognosis of NSCLC patients. Therefore, our results suggest a promising use of TrxR1 as a valuable prognostic marker in EGFR wild type, ALK negative advanced NSCLC patients, and the combined detection of TrxR1 and CEA may be able to select poor prognosis patients more accurately. However, we found that there was still no joint effect between TrxR1acitivity and CEA concentration. It needed to be further investigated.

In recent years, several serum biomarkers were found to be associated with the prognosis of NSCLC such as Cripto-1 [25], haptoglobin [27] and chemerin [28]. However, the prognostic value of those biomarkers remains to be further verified and those studies had not screened the patient’s TNM stage and driver gene status which had great influence on the patient’s treatment and survival. In our study, in order to pick out the patients with worst prognosis, we recruited EGFR wild type and ALK negative advanced NSCLC patients which did not have the opportunity to receive target therapy. In our previous study, we found serum TrxR1 activity was significantly higher in NSCLC patients than healthy control subjects. Ethaselen(BBSKE) was a novel TrxR1 targeted inhibitors discovered by State Key Laboratory of Nature and Biomimetic Drugs of Peking University and has entered the Phase I clinical trial. Our research was promising to pick out the patients with poor prognosis which we can recommend them to participate in the trials.

In summary, NSCLC patients with higher serum TrxR1 activity had poorer prognosis, suggesting that serum TrxR1 may be a useful clinical biomarkers in prognostic evaluation in EGFR wild type, ALK negative advanced NSCLC. Furthermore, combined detection of TrxR1 and CEA may have a better prognostic value.

## MATERIALS AND METHODS

### Characteristics and follow-up of data

This study was performed in Cancer Hospital of Hunan Province. From June 2013 to February 2016, a group of 142 EGFR wild type and ALK negative NSCLC patients were enrolled. Eligible patients had histologically or cytological confirmed lung cancer that was newly diagnosed. Clinic pathologic features included gender, age, histology, TNM stage (IIIB+IIIC or IV), smoking index, CEA levels, and Chemotherapy regimen (Table 1). And all patients were classified by clinical stage according to the 8th edition of AJCC TNM staging system classification. The disease was evaluated using RECIST version 1.1 criteria; tumor response was assessed by Computed Tomography imaging. And all patients’ follow-up data were from the time of first diagnosed until July 30, 2016. All patients were followed for PFS and OS. Progression-free survival (PFS) was defined as the time interval between the date of diagnosis and the date of disease relapse. Overall survival (OS) was defined as the time interval between the date of diagnosis and the date of death. This study was approved by the ethics review committee.

### The serum activity of TrxR1 is processed by ELISA

The whole bloods with heparin anticoagulant of patients were collected in hospital (5 mL each). Samples were then centrifuged at 3000 r/min, 10min at room temperature for separation. The serum was collected and stored in −80°C fridges for further test. TrxR1 activity in the serum of each sample was analyzed with enzyme-linked immunosorbent assay ELISA kits (Thioredoxin Reductase 1 kit, keaise Medicine, Weihan City, HuBei Province, China) according to the manufacturer’s instructions. A 96-well microplate was precoated with anti-human TrxR1. The standard dilution series ranged from 100 to 1, first, 100μl of each standard or serum sample (1:100) was added to appropriate wells and incubated for 2 hours at room temperature with gentle shaking. After discarding the solution and washing 3 times, 100μl of prepared biotinylated anti-human TrxR1 antibody was added to each well and incubated for 30 minutes. After washing away unbound biotinylated antibody, 50μl horseradish peroxidase (HRP)-conjugated streptavidin was pipetted into the wells and incubated for 45 minutes, and 50μl 3, 3′, 5, 5′-Tetramethylbenzidine (TMB) one-step substrate reagent was added after 5 washes. Subsequently, 50μl stop solution was added to each well, and the plate was immediately record the color development at 450nm for 10 to 45 minutes. The CEA levels were measured by electrochemiluminescence immunoassays. EGFR mutation were tested by amplification refractory mutation system (ARMS), ALK testing was performed centrally with the use of Ventana Immunohistochemistry (IHC).

### Statistical analysis

Statistical analyses were performed with SPSS 19.0 software (SPSS for Window, SPSS Inc, Chicago, IL). And the differences between groups were determined by t-test. The Progression-free survival (PFS) and Overall survival (OS) was calculated using the Kaplan-Meier method and was analyzed using the log-rank test. Survival analysis was performed using the Kaplan-Meier method and the log-rank test. Multivariate analysis was conducted to determine an independent impact on survival using the Cox proportional hazard method. For all tests, P-value<0.05 was considered as statistically significant.
